# Household Hints and Recipes

**Published:** 1877-04

**Authors:** 


### Household Hints & Recipes.

MILK.—The unpleasant taste imparted to milk and butter by feeding turnips, etc., may be removed by simply throwing into each pan of milk of four or five quarts as much saltpeter as will lie on the point of a knife.

---

PLASTIC MATERIAL.—Five parts of sifted whiting mixed with a solution of one part glue, together with a little Venice turpentine to obviate the brittleness, makes a good plastic material, which may be kneaded into figures or any desired shape. It should be kept warm while being worked. It becomes as hard as stone when dry.

---

HOT-WATER-PROOF CEMENT.

Gelatin, 5 parts.
Soluble acid chromate of lime, 1 part. Mix.

Cover the broken edges with this, press lightly together, and expose to sunlight, the effect of the latter being to render the compound insoluble even in boiling water.

---

CEMENT TO ATTACH TIN TO METALIC SUBSTANCES.

Mucilage gum tragacanth, 10 ozs.
Honey of roses, 10 ozs.
Flour, 1 oz.

Mix.

---

TO CLEAN GLOBES.—If the globes on a gas fixture are much stained on the outside by smoke, soak them in tolerably hot water, in which a little washing soda has been dissolved. Then put a teaspoonful of powdered ammonia in a pan of luke warm water, and with a hard brush scrub the globes until the smoke stains disappear. Rinse in clean cold water. They will be as white as if new.

---

INK FOR MARKING.—Instead of the nitrate of silver preparations, which of course can perhaps hardly be excelled, there are several equally good for many purposes, and for some uses better. They resist alkalies, acids, chlorine, iodine, and no menstrua will disolve them that will not also destroy the tissue on which they have been employed. They cannot be conveniently used with pens, but they are well adapted to *types* and *stencil plates.*

1. Take of genuine Trinidad asphaltum, one part; oil of turpentine, four parts; lampblack or ivory black, enough to give a strong black color. Rub together thoroughly.

LIQUID SHOE POLISH.—This formula is sent to us as an answer to the same query; it is from a German source:

Dissolve shellac, 3½ oz.

In alcohol, ½ pint.

Rub smooth.

Lampblack, 35 grs.

With cod liver oil, 6 dr.

Mix, and apply a few drops with a sponge.

---

CHROMATIZED GELATINE AS A CEMENT.—Chromatized Gelatine, obtained by the addition of one part of bichromate of potass to five parts of a solution (5 or 10 per cent.) of gelatine, forms a most excellent cement for glass. The surfaces to be united, after being smeared with the cement, are placed upon each other and exposed to the sun.—After a few hours the adhesion is perfect and almost invisible, and boiling water itself has no action upon the cement.—*Journal de Med de Bruxelles.*

---

THE REMOVAL OF GREASE SPOTS FROM MARBLE.—The German *Building News*, (*Bauzeitung*) says:—To remove grease spots from marble is no very great task—for the most part the grease penetrates deeply, and is very obstinately retained by the crystalline substance. A satisfactory result is obtained most quickly by smearing a semi-fluid paste of benzole and chalk mud, in a layer about 20 millimetres thick, over the spots, and covering with a wet cloth. The operation must be repeated until the spots disappear.—*Chemist and Druggist.*

---

RESTORING FADED INK.—Mr. Garside, of Southport, England, describes in the *Pharmaceutical Journal* a method he successfully employed for restoring the legibility of a letter which had been submerged in the wreck of the *Schiller*, and which was quite illegible until submitted to this process. The letter was carefully brushed over with solution of sulphocyanide of potassium (1 in 20), and then, still damp, held over a dish containing hot hydrochloric acid, the writing thus developed was a deep red color. The philosophy of the process is this: The iron of the ink is precipitated as peroxide upon the fibers of the paper, and remains when all other coloring matters are washed away. Being in an insoluble form, however, no effect is produced by the reagent until the fumes of the acid have rendered it soluble. Probably ferrocyanide of potassium would answer as well or better than sulphocyanide.

MILK ON CARPETS.—To remove freshly spilt ink from carpets, first take up as much as possible of the ink with a teaspoon. Then pour cold sweet milk upon the spot, and take up as before, pouring on milk until at last it becomes only slightly tinged with black. Then wash with cold water, and absorb with a cloth without too much rubbing.

---

POTTER'S CLAY FOR COAL STOVES.—Mix the clay with say one-third of fine sand and wood ashes in proportion of about one-fourth of the whole (in bulk); add common salt in a much smaller proportion, say one pound to twenty-five; the exact proportions are not essential; mix with water to a stiff dough, and line the fire-pot to the proper thickness; dry by means of a light wood fire at first, which may be built in the ash-pan below, to avoid injuring the mortar before it hardens. It will bake nearly as hard as stone. Common yellow loam will take the place of potter's clay and will not need the sand. Fire-clay is said to answer an excellent purpose with salt only.

---

EXTERMINATING BEDBUGS.—Where all other means have failed to exterminate bedbugs, sulphurous acid gas has succeeded. Take everything out of the infested room, plug up all the windows tightly, close all chimneys, and empty about 1 oz. of powdered sulphur on a pan of hot coals, placed in the middle of the floor; shut the doors and cover all cracks; let the sulphur burn as long as it will. Where the room is large, it is a good plan to fasten a bit of tin tube to the bottom of the pan, and to this connect enough small rubber pipe to lead out of the nearest door. By blowing into the end of the pipe with the bellows, the sulphur will be caused to burn more quickly by the draft created, and to give a denser smoke. After the sulphur has burned out, paint all the cracks in the floor and mop board with a strong solution of corrosive sublimate, and treat the furniture to the same before replacing it. We have seen a room frightfully infested completely freed by this plan.—*Scientific American*.

---

RED WASH FOR BRICKS.—The *Enquirer* gives the following receipt:

Venetian red and Paris brown, in proportion to suit the taste, are mixed with a quantity of water to make a heavy wash. With this the walls are well coated. To settle the color to the wall, and prevent its washing off by the rain, a wash with diluted muriatic acid (one-quarter acid) is given over the painted surface.

The mixture forming the white lines or joints is made of settled white lime, to which is slowly added plaster of paris, kept stirring until the mixture is past setting; then mix a little fine sand, to keep from cracking, and work into the consistence of glazier's putty. This putty is then applied to the walls by two men, along a straight edge, and with a beading trowel, the distance of the joints having previously been measured. Care should be taken, in applying this putty to press it strongly against the wall, to prevent any water from creeping between it and the brick. In winter time we should suppose that the water would freeze, expand, and detach the white joint, thus spoiling the looks of the building. But it is important if the treatment above described proves effectual. It is certainly worth trying, for an indestructible red and white paint on brickwork is very beautiful.

---

LIQUID SLATING FOR BLACKBOARDS.—*Abilene, Kansas* kindly communicates the following receipts, which, we doubt not, will be of service to some of the readers of the *Circular*. The first one he has sold for several years, with perfect satisfaction to those who have used it, and he recommends it as a practical working formula. The second is the preparation formerly used by a gentleman when principal of schools in the East, and said by him to be superior to all others:

Alcohol, 95 p. c., 1 gallon.
Shellac, 1 pound.
Best ivory black, 8 ounces.
Finest emery flour, 5 "
Ultramarine blue, 4 "

Make a perfect solution of the shellac in the alcohol before adding the other articles. To apply the slating, have the surface smooth and entirely free from grease; shake well the bottle containing the preparation, and pour out a small quantity only into a small dish, and apply it with a new flat varnish-brush as rapidly as possible. Keep the bottle well corked, and shake it up each time before pouring out the liquid.

---

TO COLOR IVORY BALLS.—In former years we published a way to proceed for accomplishing this purpose. Without repeating ourselves we offer you the following methods. It may be stated, however, *in limine*, that ivory can be died the same as wood, by carefully first cleaning from grease and other impeding matters. You will begin for *black* by washing the ivory in alkaline water, then immersing for a time in a weak neutral solution of nitrate of silver, and afterwards exposing it to the light; or drying and then dipping it in a weak solution of hydrosulphate of ammonia.

*Brown* may be given to the ivory by following the foregoing directions, except that the solution of silver should be considerably weaker.

For *Green*, dissovle the acetate of copper in vinegar, and allow the ivory to lie in the solution for a short time, being careful to use a glass or stoneware vessel as the vat. The same end may be attained by taking two parts of acetate of copper and one part of muriate of ammonia dissolved in soft water.

*Red.*—Make an infusion of cochineal in liquor of ammonia, then immerse the ivory after having soaked it for a few minutes in water slightly acidulated with aqua fortis.

*Purple.*—Make a weak solution of terchloride of gold, steep in it the ivory, and then expose it to the light.

Ivory may be *whitened* or bleached by placing it for a little time in water acidulated with sulphurous acid, or with chloride of lime, or chlorine in solution. All these colors can be given to ivory by using for the purpose some of the aniline preparations.

---

HOW RICE SHOULD BE COOKED.—Mr. F. B. Thurber, of New York, writing from Japan to the *American Grocer*, gives the following account of the Japanese method of cooking rice: "Rice is worth here from $1.50 to $1.75 per picul of 133 pounds, or about 1⅛ to 1⅜c. per pound; at first thought it seems as if there might be a profit to import it into the United States, but our duty of 2½c. per pound, together with freight, insurance, and premium on gold, bring it up to a figure where there is no margin. They do know how to cook rice here though, and for the benefit of grocers and customers in the United States I investigated the matter. Only just enough cold water is poured on so as to prevent the rice from burning at the bottom of the pot, which has a close-fitting cover, and with a moderate fire, the rice is steamed rather than boiled until it is nearly done; then the cover is taken off, the surplus steam and moisture allowed to escape, and the rice turns out a mass of snow-white kernels, each separate from the other, and as much superior to the usual soggy mass we usually get in the United States as a fine mealy potato is superior to the water-soaked article. I have seen something approaching this in our Southern States, but I do not think that even there they do it as skillfully as it is done here, and in the Northern States but very few persons understand how to cook rice properly. I am sure that if cooked as it is here, the consumption of this wholesome and delicious cereal would largely increase in America."

---

IN CASE OF FIRE.—The late Dr. Hall made these excellent suggestions to be followed in case of fire, and now that the season of fires is at hand the remarks are timely: Keep the doors and windows of the structure closed until the firemen come; put a wet cloth over the mouth, and get down on the hands and knees in a smoky room; open the upper part of the window to get the smoke out; if in a theatre, church, or school-room, keep cool; descend ladders with a regular step to prevent vibration. If kerosene just purchased can be made to burn in a saucer by igniting with a match, throw it away. Put wire-work or glass shades over gas-lights in show windows, and in bedrooms with curtains; sprinkle sand instead of sawdust on the floors of oil stores; keep shavings and kindling wood away from steam-boilers, and greasy rags from lofts, cupboards, boxes, etc.; see that all stovepipe enters well into the chimney, and that all lights and fires are out before retiring or leaving the place of business; keep matches in metal or earthen vessels, and out of the reach of children; and provide a piece of stout rope, long enough to reach the ground, in every chamber. Neither admit any one if the house be on fire, except police, firemen, and known neighbors; nor swing lighted gas brackets against the wall; nor leave children in a room where there are matches or an open fire; nor deposit ashes in a wooden box, or on the floor; nor use a light in examining the gas meter. Never leave clothes near the fireplace to dry; nor smoke or read in bed by candle or lamp light; nor put kindling wood to dry on top of a stove; nor take a light into a closet; nor pour out liquor near an open light; nor keep burning or other inflamable fluids in rooms where there is a fire; nor allow smoking about barns or warehouses.—*Manuf. and Builder*.

---

TREATMENT OF WINE.—In the *Wine Trade Review* of July 15, we find the following account of a method of treating wine, patented by Dr. Thudichum:

"The features of novelty of this invention are as follows: The inventor ascertains the amount of sulphuric acid contained in the wine by chemical analysis, in the manner well known in science. He then adds to the wine a quantity of tartrate of barium in the state of a very fine powder, or in the state of a freshly made precipitate; or he adds to the wine a quantity of tartaric acid first, and a quantity of hydrated caustic baryta dissolved in a minimum of hot water afterwards, sufficient in each case to precipitate nearly all the sulphuric acid and a portion of the potash, and agitates the mixture in an apparatus which he has invented for that purpose. After the lapse of a few days, he racks, decants, filters, or otherwise separates the clear wine from the precipitate. He then allows the wine to repose in a cool place for some time to enable it to deposit any surplus of tartar or bitartrate of potassium which it may contain. When clear, the wine is tested, to prove that all the impurities have been removed, and that not a trace of the agents employed in this removal has remained in the wine, excepting alone a small portion of tartaric acid. And he declares the effect of his discovery and invention to be that the wine treated by this process is much improved in taste and flavor, that its color is riper, and that it matures much better than wine not so treated, and that it has therefore a much greater money value than the impure wine containing the said impurities. And he further declares that by this method the ordinary processes of fining wines by the blood of oxen and sheep, by whites of the eggs of birds, or by cows' milk, or by Spanish earth (so called), or isinglass or gelatine, or by dextrine and sulphurous acid, or the fumes of sulphur burnt—all of which, though fining the wine, leave some of their soluble ingredients in the wine, and compromise its quality—are made unnecessary and superfluous. And he further declares that by these processes, the wines treated by them are made much more wholesome than they are in their ordinary impure state, and that therefore his discovery and invention will not only be of advantage to persons using it for profit, but will be of public utility."

---

CHEAP WALKS.—How to make walks about the house yard without much cost, is a question which we have often asked ourselves without receiving a satisfactory reply. Coal ashes, sand, such as is used for mortar, and clay in the proportion of rather more sand than coal ashes—the difference made up with the clay—all well mixed together and laid at least four inches deep, will become very compact and hard. Macadamized paths are to be recommended in point of cheapness. Collect all the stones you can. Place the large ones in the bottom and fill in with the smaller ones, covering, as above, with sand, ashes and a little clay. *Moore's Rural.*

Mrs. Henry Ward Beecher, in the *Christian Union*, gives the following:

—Benzine, ether, or soap will take out spots from silk, but remember the goods must not be rubbed.

—Soapstone hearths should be first washed in pure water, and then well sprinkled with powdered marble or soapstone, and rubbed with a piece of the same stone.

—Spots from sperm candles, stearine, and the like, should be softened and removed by ninety-five per cent. alcohol, then sponged off with a weaker alcohol and a small quaniity of ammonia added to it.

—Oil of turpentine or benzine will remove spots of paints, varnish, or pitch from white or colored cotton or woolen goods. After using it they should be washed in soap-suds.

—We know of no remedy that will restore silks injured by rust or nutgall ink. Any attempt to erase the spots which we have ever known tried only increased the evil.

—Tinware looks much nicer when washed in hot water with milk instead of soap, and will not require the rough scouring which is so commonly used by servants, and which soon wears off all the tin, leaving a rusty, useless article, neither iron nor tin.

—Colored cottons or woolens stained with wine or fruit should be wet in alcohol and ammonia, then sponged off gently—not rubbed—with alcohol; after that, if the material will warrant it, washed in tepid soap-suds. Silks may be wet with this preparation when injured by these stains.

—Roll a lemon till soft, then cut a thick slice and bind on a corn at night. If white in the morning it can be easily extracted. A very bad corn may take several applications before a cure is effected. We have never tried it, but have good authority for thinking it will effect a cure.

—Holding white cotton or linen over the fumes of burning sulphur, and wetting in warm chlorine water, will take out wine or fruit stains. The sooner the remedy is applied after any of these spots or stains are discovered the more effectual the restoration.

—When chromos require cleaning remove all dust with a feather brush, and wipe carefully with a soft chamois-skin, or fine linen cloth, very slightly dampened. If a little spotted or dull, a drop of oil on the chamois will remove it. If the varnish is dull or rubbed off, re-varnish with thin mastic varnish. Like oil paintings, it is not desirable to hang chromos in a dark room; but never expose them to the direct rays of the sun.

—Never wash mirrors with a cloth; but after removing all dust from mirror and frame with an old silk handkerchief or feather duster, dampen an old newspaper and rub the surface of the mirror till perfectly clear and free from spots; then wipe off all the moisture with a dry paper, and the mirror will be as clear as glass can be—without motes or streaks, as is the case when it is washed and polished with a cloth.

—Tea leaves may be saved from the table for a few days, and when sufficient are collected, *steep*, not *boil* them for half an hour in a tin pan; strain the water off through a sieve, and use this tea to wash all varnished paint. It removes spots, and gives a fresher, newer appearance than when soap and water is used. For white paint, take up a small quantity of whiting on a damp piece of old white flannel, and rub over the surface lightly, and it will leave the paint remarkably bright and new.

—Grease spots may be taken from white linen or cotton by soap-suds or weak lye, and from calicoes with warm soap-suds. Grease spots on woolens can be taken out by soap-suds or ammonia. On silks use either yolk of egg with water, magnesia, ether, benzine, ammonia, or French chalk. Either is good. These are mostly used by the French, who have great skill in cleaning spotted or stained fabrics. Most of them we have used, and know them to be reliable.

—In washing dishes, fill the dish-pan half full of very hot water, and put to that quantity a half cup of milk. It softens the hardest water, gives the dishes a clear, bright look, and preserves the hands from the rough skin or "chapping" which comes from the use of soap. It cleans the greasiest dishes without leaving the water covered with a greasy scum. Iron pots, saucepans, and dishes of any kind in which food is cooked, should be filled in part with hot water and set on the range as soon as the food is removed, to be kept hot till ready to wash them. This sends most of the grease from the pan into the hot water. As soon as ready to wash these pots and kettles, pour out the hot, greasy water and wash in very hot milk and water, as above directed.

—Soap and pulverized chalk spread over mildewed spots or linen, and laid in the sun, will remove the mildew without any injury to the material. The juice of a lemon added will hasten the cure. Or dissolve an ounce of oxalic acid in a quart of water; wet the spot mildewed in this solution and lay it in the sun; it will disappear in a few minutes; or hold the spot where thus wet over the steam of a boiling teakettle and it will vanish instantly. The goods must be washed, boiled, and rinsed immediately. We do not think this any more effectual in its operation than the soap and chalk with lemon juice added, and it certainly is not so safe, as it may injure the fabric, even with the greatest care, and the solution is a deadly poison—never safe where there are children about. There seems to be no place so inaccessible, no spot so secret that these "troublesome comforts" are not able to search out and invade. If oxalic acid is used, keep it closely corked, and if you can, place it too high for any infantile aspirant to reach or climb to.

—Eider-down skirts and quilts are a luxury too expensive to be indulged in where economy is necessary; but what we are told is an excellent substitute, and in some respects superior, can be had by almost any one, yet is thrown away or wasted for the lack of knowledge. Take the coarser of tame or wild fowl, such as no one thinks of saving, and cut the feather part from the quill with scissors. Do not strip it off; that would take the thin outer skin of the stem, which when dry would become stiff and hard like wire; but run the scissors along pretty close to the quill, and clip off the plumy part. Put it into a bag, and hang it up in a cool, airy place, where it can be kept perfectly dry. When the bag is full, but not crowded, tie it up closely, and, laying it on the table, begin to work the plumes inside into a mass almost like a sheet of cotton wadding. Pull them, knead them, shake them all together till you can feel, through the tick, that they have become a united mass. This will form a downy, felt-like fabric, wonderfully light. It is a French economy, and superior to the natural down in this respect: that having no stems it cannot work through the outside cover of the garment or comfortable and float like snowflakes over the room, but keeps its place as surely as wool or cotton batting, is much lighter and warmer and costs nothing but the time, which any child can give and call it play.

---

REMOVING MILDEW.—To take mildew from linen, mix soft soap with powdered starch, half the quantity of salt and a piece of lemon, and lay it on both sides with a paint brush. Let it be in the open air—on grass is preferable—till the stain is removed.

